# The Role of Glutathione Metabolism in Chronic Illness Development and Its Potential Use as a Novel Therapeutic Target

**DOI:** 10.7759/cureus.29696

**Published:** 2022-09-28

**Authors:** Boris D Hristov

**Affiliations:** 1 Surgical Critical Care and Acute Care Surgery, Brookdale University Hospital Medical Center, New York, USA

**Keywords:** atherosclerotic cardiovascular disease, neurodegenerative disesase, chronic liver disease (cld), chronic renal disease, metabolic syndrome, ggt, n-acetylcysteine, glutathione

## Abstract

Glutathione (GSH) is the most abundant thiol antioxidant in the human body and serves many important biochemical functions, including the regulation of vitamins, such as vitamins D, E, and C, and detoxification of drugs and toxins. As a powerful antioxidant, GSH is particularly important as a regulator of mitochondrial metabolism and a free radical scavenger that limits oxidative damage to cellular components. Low GSH levels have been associated with many chronic pro-inflammatory conditions, such as metabolic syndrome, cardiovascular, renal, and hepatic disease, as well as neurodegenerative conditions and autoimmune diseases. Given GSH’s known direct protective role in mitochondrial metabolism and its association with chronic diseases of highly metabolically active tissues, this review aims to examine the literature for evidence that low GSH levels may be an important causative factor in the development of chronic illnesses. Because no large prospective human trials have been conducted using direct measurements of GSH, this review focused on the more common biomarker gamma-glutamyl transferase (GGT) which is directly correlated to low GSH levels. Several large prospective studies support this hypothesis by demonstrating that higher GGT levels are correlated with the risk of developing metabolic syndrome and cardiovascular disease in a dose-dependent fashion. Furthermore, as a corollary to this hypothesis, human and animal trials utilizing GSH augmentation using precursor supplementation in chronic conditions, including metabolic syndrome, cardiovascular disease, hepatic disease, renal disease, and neurodegenerative conditions, were also reviewed. While many of these trials were preliminary and small, there is strong evidence that GSH supplementation leads to improved outcomes in all of these chronic conditions. This review seeks to highlight these studies as preliminary evidence demonstrating the contributory role of GSH in chronic disease progression because a simple and cost-effective strategy can be created to screen for, track, and intervene in susceptible patients in the primary care setting at the earliest possible time in the disease process. Such a novel strategy would impact the majority of chronic diseases contributing to the bulk of morbidity and mortality in the Western world, and, thus, even minor benefits across many conditions may substantially impact population-wide health and longevity.

## Introduction and background

Glutathione (GSH) is a tripeptide molecule consisting of the amino acids glutamate, cysteine, and glycine. It is the most abundant thiol-containing antioxidant in the human body and exists in both a reduced and oxidized state in cells, with over 95% of GSH existing in its reduced state [1]. GSH was initially isolated from yeast in 1888 by J. de Rey-Paihade and subsequently found in animal tissues by Frederick Gowland Hopkins in 1921. Hopkins was also the first to identify the constituent amino acids of GSH in 1929 and was corroborated independently by Edward Calvin Kendall.

Subsequent research on the biological role of GSH has shown that it serves a critical role in protecting cells from oxidative damage by neutralizing damaging reactive oxidative species (ROS) in cells by reducing them before they can damage critical cellular components such as DNA. This antioxidant function plays an especially important role in mitochondria which generate ROS through the natural function of the electron transport chain, particularly during periods of metabolic stress. Human studies support the clinical importance of this relationship by demonstrating that an increased ratio of oxidized to reduced GSH is closely correlated with global levels of oxidative stress and inflammation [2]. Similarly, this is also seen in the setting of many chronic diseases such as metabolic syndrome and diabetes [3,4], atherosclerosis and cardiovascular disease [5], chronic kidney disease [6], neurodegenerative conditions such as Alzheimer’s disease and Parkinson’s disease [7-9], as well as autoimmune diseases [10], cancer [11] and chronic infections such as human immunodeficiency virus [12].

GSH also plays an important role in the detoxification of toxins, xenobiotics, and drugs. Decreased levels of GSH have been noted in alcoholics, smokers, and patients with chronic liver disease [13]. It is particularly important in acetaminophen overdoses where increasing GSH levels by supplementing the precursor N-acetylcysteine (NAC) is the treatment of choice to clear the toxic intermediate metabolite N-acetyl-p-benzoquinone imine (NAPQI) via conjugation [14]. Unconjugated NAPQI which overwhelms the capability of endogenous GSH to reduce it can bind to cellular proteins damaging them in the process and inducing cell death [15]. Other important functions attributed to GSH include the regulation of immunity, the synthesis of leukotrienes and prostaglandins [16,17], and the regulation of vitamin D, E, and C metabolism [18,19]. Moreover, it plays an important role in glutamate neurotransmitter metabolism and neural regulation [20]. Because GSH is implicated in several biochemical processes and regulates other antioxidants, it is often referred to as the “master antioxidant.”

## Review

The biochemical role of glutathione as a driver of chronic illness

Low total GSH levels and elevated ratios of oxidized to reduced GSH are common in chronic illnesses as well as advanced age. While these relationships have been known for years, most literature has overlooked these findings as the predictable result of increased inflammation and oxidative stress similar to other biomarkers such as C-reactive protein (CRP). Few, if any, studies have explored the possibility of low GSH levels as a potentially important causative driver of disease pathology in itself [21]. However, due to the important biochemical role of GSH in protecting mitochondria from oxidative damage, this link should be re-examined. GSH protects mitochondria via the glutathione-ascorbate cycle, also known as the Asada-Halliwell pathway [22], which is the main pathway to neutralize the hydrogen peroxide radicals created as a byproduct of normal metabolism. Through this pathway, electrons are channeled from nicotinamide adenine dinucleotide phosphate (NADPH) to reduce hydrogen peroxide, with GSH as the rate-limiting step for regenerating NADPH [23,24].

Without adequate GSH to replenish NADPH, low NADPH levels result directly in increased ROS which can damage mitochondria, DNA, and other important cellular components [24,25]. This process is most pronounced in metabolically stressed cells, and the accelerated damage to these organelles can lead to premature dysfunction and early cell senescence, culminating in cell death [24]. Thus, in the most metabolically active tissues such as renal cells, hepatocytes, endocrine cells, endothelial cells, and neurons, low GSH levels should be expected to be directly correlated with worse cellular function and early cell death. In essence, the biochemical role of GSH in mitochondria is analogous to motor oil in a gasoline combustion engine, and it is a key protector of the cellular powerhouses from the damage caused by everyday metabolic demands. Without its protection, one can reasonably expect a diminished useful lifespan in many of the critical tissues which are necessary to support normal homeostasis.

The underlying biochemistry implicates low GSH as an important contributing factor in the development of several chronic illnesses that have been associated with increased oxidative stress such as metabolic syndrome, kidney disease, neurodegenerative diseases, liver disease, and cardiovascular diseases. Fundamentally, all of these conditions can be at least partially attributed to the dysfunction and failure of mitochondria in different highly metabolic tissues. This implies that GSH levels may be used as a useful predictive marker to identify patients who have an elevated risk of developing these chronic conditions and may also offer a novel way to delay or even outright arrest disease progression by replenishing endogenous GSH using simple dietary modifications and lifestyle changes at the earliest possible stage when such interventions are most beneficial. Interestingly, there is evidence in the literature that both of these hypotheses are true.

Evidence that glutathione metabolism can predict chronic disease development and mortality

Currently, there is very limited population-wide data on the potential predictive value of GSH levels because it is not a common clinical test and measuring the ratio of oxidized to reduced GSH in the body is time-consuming and expensive. The evidence for the possible predictive nature of GSH is seen in large studies utilizing the more common biomarker gamma-glutamyl transferase (GGT) and its correlation with increased all-cause mortality. GGT is a well-known biomarker typically associated with hepatocyte damage. Biochemically, GGT is associated directly with GSH regulation and maintenance of its normal redox status in cells by breaking down extracellular GSH which cannot cross the cellular membrane to release cysteine and glycine building blocks which can more easily cross into the cell for de-novo synthesis of GSH during periods of oxidative stress [26].

**Figure 1:**
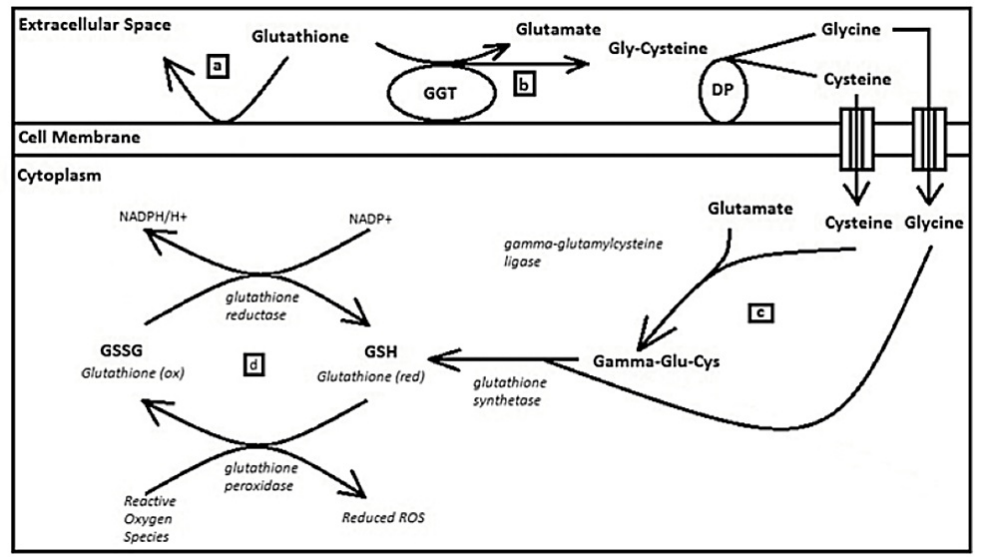
Extracellular glutathione is unable to cross the cell membrane as it is not lipophilic (a). In times of increased oxidative stress which may outstrip the ability of intracellular stores of glutathione to reduce all reactive oxygen species produced during metabolism, the cell will increase expression of gamma-glutamyl transferase bound to the cell membrane (b) as this enzyme will readily break down extracellular glutathione in unison with membrane-bound dipeptidases to its constituent amino acids glutamate, cysteine, and glycine. These amino acids readily cross the cell membrane and serve as building blocks for glutathione synthesis (c). Once new glutathione is synthesized, it can be used to reduce the damaging reactive oxygen species (d). GSH: glutathione; GGT: gamma-glutamyl transferase

Thus, elevations in GGT on the external cellular membrane are correlated directly to relative GSH deficiency in the cellular cytoplasm of the host cell. Furthermore, while this marker is traditionally associated with hepatocytes, studies have shown that this marker is elevated in cardiovascular disease and chronic kidney disease [27-29]. Experimentally, this relationship has been verified in breast cancer patients with an r^2^ of 0.6262 [30], supporting its use as a surrogate marker of low global GSH states in general.

Examining numerous liver function tests collected in the life insurance industry, Pinkham et al. showed that GGT levels had one of the best predictive dose-dependent negative correlations with all-cause mortality among a group of 560,000 life insurance applicants whose primary cause of death was cardiovascular disease [31]. In another study of life insurance data, Palmier et al. looked specifically at all-cause cancer mortality using a multivariate analysis of biochemical markers from life insurance applicants and found that liver function tests, specifically GGT and alkaline phosphatase (ALP) were most predictive of all-cause cancer mortality [32]. In their analysis, the hazard ratio (HR) for cancer mortality in 40-49-year-old males nearly doubled from 0.75 in the population with GGT levels in the 15-20 U/L range to about 1.45 in the patients with GGT levels in the 45-85 U/L range. While both of these studies were retrospective, the predictive utility of GGT has been demonstrated in prospective trials as well. In a trial of 3,124 subjects free of liver disease who were followed for an average of 40 months, Monami et al. showed that elevations in GGT over 40 U/L were associated with increased incidence of diabetes diagnosis (HR = 2.54 (1.26-5.11); p = 0.05) and cardiovascular disease diagnosis (HR = 2.21 (0.98-5.43); p = 0.10) [33]. Lee et al. corroborated these findings in more detail in the Framingham Heart Study which examined 3,451 participants over 19 years who were stratified into four groups based on GGT level. The lowest quartile of patients had GGT values between 1 U/L and 11 U/L in men and 1 U/L and 6 U/L in women, and the highest quartile included men with GGT values from 25 U/L to 99 U/L and women with GGT values from 14 U/L to 88 U/L. In this study, groups with higher GGT levels showed dose-dependent elevations in all-cause mortality and cardiovascular disease when adjusted for age, sex, body mass index (BMI), blood pressure, alcohol consumption, and smoking. The quartile cohort with the highest GGT elevation had a 23.8% incidence of cardiovascular disease compared to the lowest quartile which had a 10.5% incidence (HR = 2.11). For all-cause mortality, the incidence was noted to be 16.1% in the highest quartile group compared to 6.3% in the lowest quartile group (HR = 2.21) [27]. Because GGT elevations correlated in a relatively tight and dose-dependent relationship with the outcomes tracked in both of these studies, these findings are suggestive of an underlying mechanism of causation.

Evidence that glutathione augmentation can delay or reverse chronic illness progression

Whereas population-wide studies tracking GGT illustrate the predictive potential for chronic diseases and mortality of decreased GSH levels, other studies utilizing NAC to increase cellular GSH stores have demonstrated that it is possible to intervene and have meaningful improvements in the same conditions. Oral GSH has extremely poor oral availability and is not lipophilic to easily cross the cell membrane and thus cannot be used to effectively increase systemic intracellular GSH levels. Instead, precursor supplementation with the rate-limiting precursor amino acid building block NAC is typically used in studies to increase endogenous GSH levels. NAC has been validated experimentally to increase GSH levels and redox homeostasis in patients with GSH deficiency [34] and is often combined with glycine (another GSH precursor amino acid) in some study protocols for enhanced efficacy.

In liver disease, NAC administration was first utilized and proven efficacious in acute conditions. It was first suggested as a possible treatment for acetaminophen toxicity in 1974 [35], and its efficacy was demonstrated by Smilkstein et al. in 1988 in a landmark trial that used oral NAC in 2,540 patients suffering from acetaminophen poisoning [36]. The study showed zero mortality, improved hepatic function, and faster recovery in patients who began treatment within 16 hours of acetaminophen ingestion. In more recent studies, the benefits of NAC have been demonstrated in acute liver failure from non-acetaminophen causes, including viral infection and other drugs [37]. Furthermore, NAC administration has been associated with improved transplant-free survival in liver transplant patients [38], possibly by attenuating the damage from ischemia and reperfusion of the grafts.

The benefits of NAC administration in heterogeneous causes of acute liver failure demonstrate that GSH augmentation can improve clinical outcomes in diverse conditions that lead to hepatic oxidative stress and not just acute acetaminophen toxicity. Analogously, improved outcomes should extend to more chronic inflammatory conditions as well. Indeed, using a rat model, Ozaras et al. demonstrated that supplementation with NAC in animals undergoing oxidative stress from ethanol infusions led to decreased inflammatory markers such as aspartate aminotransferase (AST), alanine transaminase (ALT), and GGT dramatically [39]. In this study, the decreases in the markers approached the levels of the control group without any alcohol infusion. Human trials of NAC in chronic liver disease are sparse. However, two small studies in patients with liver disease from chronic hepatitis B infection and non-alcoholic fatty liver disease reported some benefits. In 90 patients with acute-on-chronic hepatitis B infections, Wang et al. were able to show decreases in bilirubin, GGT, and ALP and improved coagulation profiles as well as decreased intrahepatic cholestasis in the treatment group [40]. Furthermore, Khoshbaten et al. followed 15 patients with non-alcoholic fatty liver disease for three months and showed a decrease in ALT in the treatment group and, more importantly, a significant decrease in spleen size which was correlated with the degree of fatty infiltration of the liver [41].

Metabolic syndrome is another chronic condition in which NAC administration has been shown to be beneficial. An open-label pilot study by Rani et al. utilized a six-month course of NAC supplementation in diabetic patients and showed beneficial effects, including improved glucose control via decreased HbA1C, decreased blood pressure, decreased CRP, decreased triglycerides, and increase in high-density lipoprotein compared to control group patients [42]. Another pilot study by Kumar et al. using NAC and glycine supplementation for 24 weeks in eight geriatric patients reported decreased insulin resistance, body fat, and waist circumference [43]. This study also demonstrated decreases in oxidative stress levels and endothelial dysfunction, exercise strength, and cognition in the intervention group. Some of the biochemical mechanisms underlying these improvements were experimentally shown by Alnahdi et al. in vitro to be due to the minimization of the glucolipotoxicity effects and mitochondrial dysfunction caused by hyperglycemia in pancreatic beta cells [44].

Although no large human studies have been undertaken to examine the effects of NAC on the cardiovascular system long term, there are human randomized trials showing a decrease in ischemic damage by 60% after myocardial infarction in patients undergoing percutaneous coronary intervention for acute ST-elevation myocardial infarction in a randomized control trial by Pasupathy et al. [45]. A small pilot randomized control study by Marian et al. showed small improvements in measures of cardiac function in 24 patients with hypertrophic cardiomyopathy who were given NAC for 12 months compared to a control group of 11 patients [46]. However, the results of this study were not statistically significant as the study was underpowered. Other studies in animals have provided strong evidence of the potential benefits of NAC on heart disease. In a rabbit model of heart failure induced by doxorubicin administration of NAC and glycine, Wu et al. demonstrated increased GSH levels as well as decreased nuclear factor kappa B activity corresponding with overall decreased oxidative stress on the cardiomyocytes and decreased myocyte apoptosis [47]. More importantly, these findings corresponded clinically with improved cardiac function in vivo, as assessed by echocardiography. In a mice study looking at NAC supplementation in atherosclerosis, Cui et al were able to show decreased in-vivo oxidation of low-density lipoprotein in the experimental group which clinically corresponded with atherosclerotic plaque formation [48].

In renal disease, most trials utilizing NAC have typically focused on minimizing acute kidney injury due to contrast-induced nephropathy (CIN). Overall, these studies have reported mixed results, with a recent meta-analysis of 86 randomized controlled trials by Subramanian et al. showing that there may be a small benefit of low-dose NAC administration on CIN [49]. In chronic hemodialysis patients, NAC administration has been shown to decrease circulating homocysteine levels [50,51] as well as decrease other inflammatory markers, including asymmetric dimethylarginine and malondialdehyde. Other benefits noted in these studies include improved renal anemia [51,52]. Interestingly, Tsai et al. showed that NAC administration decreased homocysteine levels the greatest in patients with some preserved renal function and had minimal effects in completely anuric patients, which supports that the beneficial effects seen are due to improvement in nephron function [50]. This was corroborated by Ahmadi et al. who showed improvements in residual glomerular filtration rate in a separate randomized control trial [53]. In animal studies, NAC administration also has shown beneficial immune-modulatory effects on lymphocytes [54] and decreased interstitial fibrosis and reperfusion injury in mice models [55].

Neurodegenerative conditions such as Alzheimer’s and Parkinson’s disease are associated with neurofibrillary tangles and associated inflammatory oxidative stress. Small randomized controlled studies have shown benefits in both of these diseases. In patients with Parkinson’s disease, small randomized controlled trials of NAC and cysteine supplementation have shown an increase in dopamine binding in the caudate and putamen, as measured by ioflupane imaging [56]. In patients with Alzheimer’s disease, supplementation with NAC has been correlated with improved measures of cognitive function after three and six months on standardized assessments [57]. The neuroprotective effect of NAC has been also illustrated in rat models showing that it downregulates inflammatory markers and upregulates expression of the protective gene *Sirtuin 1* [58].

Interestingly, the neuroprotective effects of NAC are also associated with notable benefits in some chronic psychiatric conditions which are known to be associated with low GSH levels such as schizophrenia and autism. In patients with schizophrenia, randomized trials of NAC administration have shown decreases in the Positive and Negative Syndrome Scale total score, negative symptom factor, and disorganized thought factor compared to the placebo group [59]. In autism spectrum disorders, a meta-analysis by Lee et al. of four trials showed overall decreases in hyperactivity and irritability and increases in social awareness in children treated with NAC [60].

Overall, studies utilizing NAC supplementation for chronic illnesses are few and tend to be smaller pilot trials. Nevertheless, when taken together, this body of literature provides the strongest evidence that GSH plays a key role in many disparate conditions which may seem unrelated at first glance. More importantly, the evidence supports that augmenting GSH levels can lead to improved outcomes and presents a novel adjunct treatment modality that is currently not being utilized.

Potential use of glutathione metabolism in patient care

Although GSH metabolism is not directly monitored in primary care as a marker for health, the biochemical function of this molecule combined with supporting evidence from NAC trials suggests that it should. The best study supporting this assertion is by Kumar et al. [43]. This small randomized trial of elderly patients who were supplemented with NAC and glycine showed improvements in baseline insulin resistance, cognitive performance, endothelial dysfunction, muscle strength, and mitochondrial oxidative stress. While this study demonstrated the potential benefits of GSH augmentation in an elderly cohort, a future study design utilizing direct or indirect GSH level measurement in larger and more heterogeneous populations could be undertaken to more precisely identify which patients may benefit from such therapy while sparing others who may have sufficient levels of GSH from unnecessary intervention. Similar randomized controlled trials looking at vitamin D supplementation in critically ill patients have demonstrated a survival benefit in select patients who were deficient on admission, but not in patients with normal levels at the time of admission [61].

Ultimately, additional studies would need to be undertaken to identify how to optimize screening and interventions for patients as well as to provide larger population-wide data on total GSH levels, reduced to oxidized GSH levels, as well as GGT. This would no doubt necessitate many longer-term, population-wide studies. However, by examining GSH metabolism in a new way, we may be able to identify chronic diseases at the earliest possible stage of development and intervene with low-cost strategies when there is the greatest potential impact to slow down their progression. Because the chronic diseases associated with GSH deficiency are also the leading causes of death today, it serves to reason that even small potential health benefits across many chronic illnesses would have a meaningful impact in decreasing population-wide morbidity and mortality.

## Conclusions

GSH is a key thiol antioxidant in the human body which, among its many functions, serves as a major mitochondrial protector, and through this function is linked to many chronic illnesses which make up the bulk of the healthcare burden in Western societies today. Studies presented in this review show that low GSH levels have a demonstrable correlation to the faster onset of these chronic diseases and increased mortality. Fortunately, other studies have reported that GSH levels can be easily augmented in patients with elevated metabolic stress states by supplementing with its precursor-building amino acids NAC and glycine. These studies have also shown measurable clinical benefits in the study participants across many different and unrelated conditions. This review aimed to present this literature because GSH metabolism can present a novel target to identify and treat high-risk patients at the very earliest stage of the disease development process with a simple, safe, and cheap intervention. If large future randomized controlled trials can confirm these findings and a population-wide algorithm could be created and applied, then the cumulative benefits across many conditions could be dramatic even if the measurable benefits of treatment are relatively small.
